# Food availability and food access in rural agricultural communities: use of mixed methods

**DOI:** 10.1186/s12889-018-5547-x

**Published:** 2018-05-16

**Authors:** Linda K. Ko, Cassandra Enzler, Cynthia K. Perry, Edgar Rodriguez, Norma Mariscal, Sandra Linde, Catherine Duggan

**Affiliations:** 1 Division of Public Health Sciences, Fred Hutchinson Cancer Research Center, 1100 Fairview Ave. N, M3-B232, Seattle, WA 98109-1024 USA; 2 Department of Health Services, University of Washington School of Public Health, Seattle, WA USA; 3 School of Nursing, Oregon Health & Science University, Portland, OR USA; 4 Astria Sunnyside Hospital, Sunnyside, WA USA

**Keywords:** Food environment, Rural communities, Hispanics, Mixed methods

## Abstract

**Background:**

Hispanics bear some of the highest burden of the obesity epidemic and the disparities gap is bigger among Hispanics in rural communities. This mixed methods study examined the objective and subjective assessment of food availability and food access in four rural, agricultural, and predominantly Hispanic communities.

**Methods:**

In this convergent parallel mixed methods study, we used the Nutrition Environment Measures Survey (NEMS) of Food Stores and Restaurants to objectively assess 57 food stores and 69 restaurants in four rural agricultural communities in Washington State. To complement the objective assessment findings, we conducted semi-structured interviews with 32 community residents. The data were collected from 2013 to 2014. Frequencies and means were calculated for quantitative data and content analysis conducted for interview data.

**Results:**

Participants (*n* = 32) had a mean age of 35.6 (SD 6.2) years, were mostly women, uninsured, low income, and had less than a high school education. Grocery and convenience stores had low NEMS composite scores indicating low overall availability of food items, low quality, and high food prices. Composite scores for sit-down restaurants, fast casual restaurants, and fast-food restaurants were similarly low in all four towns indicating limited availability of healthier options. Semi-structured interviews revealed participants perceived high availability and accessibility of quality fresh produce. Most participants reported eating out regularly several times a week, frequenting restaurant chains that serve buffets or fast foods, and allowing children to make decisions regarding their own food choices.

**Conclusions:**

Community members’ perception of food availability and food access may be different from the objective assessment of food environment. This information can be used to inform community-wide interventions to address food environment in these rural communities.

## Background

Overweight and obesity are associated with chronic health conditions such as hypertension, diabetes, cardiovascular disease, and cancer [1]. In the United States, overweight and obesity rates have steadily increased since 1999 [2]. Hispanics bear some of the highest burden of the obesity epidemic and the disparities gap is bigger among Hispanics in rural communities [3]. At the same time, the United States has experienced a significant change in the food environment in the past several decades [4, 5], and the food environment has emerged as a powerful influence on individuals’ eating patterns, food choices, and diet quality. Food establishments have steadily increased in number resulting in greater availability of processed and convenience foods, and portion sizes have become larger in chain restaurants, fast food outlets, and food stores [5]. Simultaneously, the number of meals eaten outside the home has also increased, resulting in individuals choosing energy dense foods [6–8]. Food environment studies have examined individuals’ access to food using proximity of their homes to the nearest food store, or density of type of food stores in their communities [9, 10]. These studies characterized aspects of the food environments that exist within communities. However, a major limitation of these studies is that their findings assumed that all restaurants or food stores of the same type offered the same diet quality, health promotion information, and pricing [11]. Conversely, studies that examined characteristics of restaurants within the food environment reported variability in the proportion of healthy menu options [12, 13].

Studies on food environment helped highlight the extent of disparities that exist in resource-limited communities such as rural communities [14–16] and gave way to the rise of the term ‘food desert.’ [17] However, food availability and food access in rural agricultural communities has been less studied. Specifically, studies examining the availability of healthful foods in rural agricultural communities and community residents’ perception of food availability and access are limited. The goal of this study was to investigate the food environment and food access in four rural agricultural communities using mixed methods: quantitative nutrition environmental assessments of food outlets (food stores and restaurants) and qualitative semi-structured, in-depth interviews with community residents. Both quantitative and qualitative approaches are appropriate in this study as the use of quantitative assessments will reveal the food type and extent of healthy options available in the environment. Qualitative, semi-structured interviews will complement the quantitative findings by providing insight into individuals’ perceptions of and experiences with their food environment.

## Methods

### Study design

We conducted a convergent parallel mixed methods study in four rural agricultural communities in Eastern Washington to (1) quantitatively assess food environments using the Nutrition Environmental Measures Survey (NEMS) and (2) qualitatively assess the perception of food environments among residents. In convergent parallel design, the purpose is to gather complementary qualitative and quantitative data that provide different perspectives on the same topic, in this case, the food environment [18, 19]. We used this design to understand in what ways the objective food environment and the perception of the food environment among residents converged [18, 19]. This study was approved by the Institutional Review Board and the Ethics Committee of the Fred Hutchinson Cancer Research Center (IRB # 7890). Written consent was obtained from all the study participants prior to participation.

### Quantitative assessment

#### Setting

**Fig. 1:**
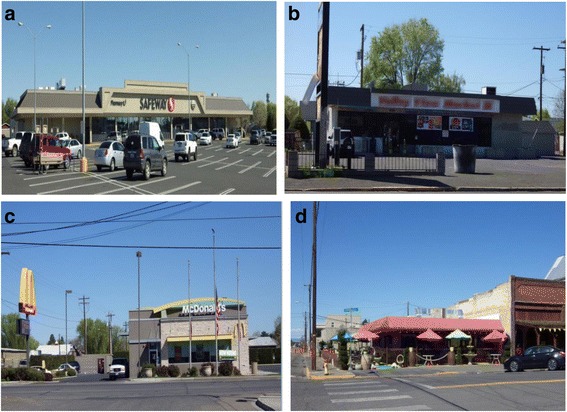
Examples of food stores in town B. **a** Grocery Store; **b** Convenience Store; **c** Sit-down Restaurant; **d** Fast Food Restaurant (Source: Author Linda Ko)

Food stores and restaurants were included if they were within a one-mile radius from one of the four designated town centers. Distance was determined using Google Earth software. Due to the small size of the towns, most food stores and restaurants fell within the one-mile radius. Therefore, our assessment included information on most food outlets in these four communities. Restaurants and stores were classified according to the NEMS protocols as sit-down, fast casual, fast food, or specialty restaurants, and grocery or convenience stores [11, 20]. Community health workers (CHW) trained in data collection using the NEMS assessment tool approached the owners or the managers of the food outlets for permission before audits took place. We approached 57 food stores and 70 restaurants; only 1 restaurant owner declined participation (Fig. 1).

#### NEMS assessment tool and scoring

Between July and November 2013, the Nutrition Environment Measures Survey for Restaurants (NEMS-R) was used to assess restaurants and the Nutrition Environment Measures Survey for Stores (NEMS-S) was used to assess grocery and convenience stores [11, 20].

The NEMS-R was designed to assess the “relative healthfulness” of food and beverage options for main menus and children’s menus, and factors that may facilitate or challenge healthful eating. The assessment consists of a menu review, an observational restaurant visit, and interviews with restaurant staff. The instrument assesses the availability of entrees, main dish salads, side dishes (i.e., fruit without added sugar, non-fried vegetables without sauce or toppings, baked chips, whole grain bread) and beverages (i.e., diet soda, 100% fruit juice, and 1% or nonfat milk) that meet the criteria for being designated as “healthy.” Guidelines for “healthy” designation were determined by federal recommendations as established by the U.S. Food and Drug Administration, U.S. Department of Health and Human Services, and the U.S. Department of Agriculture [21]. In addition, the instrument includes measures of barriers and facilitators of healthful eating and a measure of pricing and signage related to promotion of healthier or less healthy foods. Restaurant scoring was based on 8 categories: 1) sources of information, 2) site visit information, 3) site visit menu review, 4) online menu review, 5) facilitators and support, 6) barriers, 7) pricing, and 8) children’s menu. The total possible composite score for food environment quality of NEMS-R was 72 points (range − 8 to 72), where greater points indicated availability of healthier options, promotion of healthier options for adults and children, and affordable pricing.

The NEMS-S was designed to assess the availability, price, and quality of 11 indicators of food categories and healthier options, which are noted in parenthesis: fruit (fresh), vegetables (fresh), milk (skim/low-fat), ground beef (lean), hot dogs (lean), frozen dinners (reduced-calorie), baked goods (low-fat), beverages (diet soda, 100% juice), bread (whole grain), chips (baked), and cereal (high fiber) [11, 20]. Measures for each indicator include availability of healthier options, with more points if additional varieties of the healthier option were available, if quality of produce was greater (acceptable/unacceptable based on over ripeness/bruising), and if price was lower for healthy items compared to non-healthy items. Lower prices for healthier items were scored positively while points were deducted for higher prices for healthy items. Scores for availability, quality, and price were summed up to generate a composite score of food environment quality ranging from − 8 to 62 with higher scores indicating greater availability of healthy options, higher quality food products, and affordable pricing. The maximum possible score was 34 points for availability, 22 points for price, and 6 points for quality.

#### Data collection

We applied the NEMS training protocol created by the developers [11, 20] to train two CHWs in the use of the NEMS tools. The CHWs received both didactic and hands-on training, first in a classroom setting and subsequently in restaurant and store settings [22]. The first training served to provide information on the use of the NEMS tools. The second training reinforced information from the prior training to ensure high inter-rater reliability between the CHWs. After the first training, CHWs applied the learned skills by assessing two stores and two restaurants independently. A follow-up training addressed the discrepancies in the scores between the two CHWs. After the second training, CHWs independently rated two additional stores and restaurants and an inter-rater reliability of 95% was achieved. The study was approved by the Institutional Review Board at the Fred Hutchinson Cancer Research Center.

#### Data analysis

Data from NEMS were entered into SPSS version 21 (IBM Corporation, Armonk, New York) and analyzed in 2015. Frequencies were computed to describe restaurant and food store characteristics. The average composite scores of restaurants and food stores were calculated in excel spreadsheets provided by the NEMS developers [11, 20].

### Qualitative assessment

#### Study sample

The study team recruited participants through ongoing community health fairs, events for social services, and school events where NEMS assessment took place. A bilingual CHW explained the purpose of the study to the potential participants. Those who were interested provided their contact information. As this study was part of a larger childhood obesity intervention study, participants were eligible if they were parents of elementary school students in one of the four rural farming communities where this study took place.

Two bilingual CHWs experienced in qualitative approaches interviewed participants in their homes between July 2014 and November 2014. Interviews lasted between 30 and 90 min and were conducted in the participants’ preferred language, either Spanish or English. Interview questions were designed to explore participants’ and their families’ perceptions of their food environment as well as access to and use of the food stores and restaurants. Examples of questions include “How easy or difficult is it to buy fresh fruits and vegetables in your neighborhood?” “What is the quality of the produce in your neighborhood?” “How are decisions made about where you shop for food?” “What kind of restaurants do you go to when you eat out?” “Who decides what your kids order?” Prior to the interviews, the questions were reviewed by the bilingual CHWs and suggestions were incorporated to the final interview guide. Interviews were audio recorded, transcribed verbatim, checked for accuracy, and translated from Spanish into English.

#### Data analysis

Two coders independently reviewed each transcript to identify main ideas and meaning. Through manual coding, they applied a conventional content analysis approach by developing codes to capture the essence of each idea. Throughout the analysis, coders compared notes and reviewed the data, organizing findings into four themes: perceived accessibility of fruits and vegetables, perceived quality of produce, food store preference, and familial behavior around eating out. Data were analyzed January–March 2016.

## Results

### Characteristics of food Stores in Rural Communities

We collected information on 57 food stores and 69 restaurants in four rural towns: 12 grocery stores, 42 convenience stores, and 3 food stores classified as other (Table 1). Each of the four towns had more convenience stores than grocery stores. Town D had more grocery stores (*n* = 5) than any of the other towns (Town A: 1; Town B: 3; Town C: 3). Town C had the largest population and greatest number of convenience stores (*n* = 18). There were 69 eateries across all four towns. Town C had the most sit-down restaurants (*n* = 14). In Town D, fast food restaurants (*n* = 8) outnumbered sit-down restaurants (*n* = 6).

**Table 1:** Town characteristics of four rural agricultural communities

	Town A	Town B	Town C	Town D
Town Population	3246	8949	15,858	10,862
Population Density (per square mile)	1813.4	4281.8	2391.9	1743.5
Town Area (square miles)	1.8	2.09	6.63	6.31
Number of Grocery Stores	1	3	3	5
Number of Convenience Stores	3	11	18	10
Other Stores^a^	0	0	2	1
Number of Sit-down Restaurants	1	7	14	6
Number of Fast Causal Restaurants	1	3	7	2
Number of Fast Food Restaurants	0	6	12	8
Specialty Foods^b^	0	0	1	1

^a^Other stores include coffee shop and bakery

^b^Specialty foods include coffee shop and bakery

Food stores located within one-mile radius from the town centers using Google Earth software. Data collected July–November 2013

### Assessment of food stores and restaurants

Across all four towns, grocery stores had higher composite scores than convenience stores (Table 2) as well as higher scores on availability of healthy options and food product quality. Only 38 out of 57 (66.7%) food stores were stocked with fresh fruits and vegetables, and of those, only 19 stores (50%) had half of their produce rated as being acceptable in quality. Price scores of food products in grocery stores were inconsistent across all four towns. In Town B, the grocery store price score indicated that produce was more expensive than in convenience stores. Overall, both grocery and convenience stores had much lower composite scores than the maximum possible score of 62, and the price scores were similarly low among grocery stores and convenience stores.

**Table 2:** NEMS composite scores of stores by food store types in all four towns

		Town A		Town B		Town C		Town D	
	Maximum Possible Score	Grocery (*n* = 1)	Convenience (n = 3)	Grocery (*n* = 3)	Convenience (*n* = 11)	Grocery (n = 3)	Convenience (n = 18)	Grocery (n = 5)	Convenience (*n* = 10)
Availability	34.0	9.0	3.3	20.7	3.6	28.7	3.3	14.2	3.0
Price	22.0	2.0	0.2	−1.0	0.1	0.7	0.3	0.6	0.0
Quality	6.0	4.0	0.1	4.7	0.3	6.0	0.0	3.8	0.0
Composite	62.0	15.0	3.6	24.3	3.9	35.3	3.6	18.6	3.0

*NEMS* Nutrition Environment Measures Survey

The tool for NEMS Stores are from Glanz et al. [20]

Data collected July–November 2013

Composite scores for sit-down restaurants, fast causal restaurants, and fast food restaurants were similarly low in all four towns (Table 3). In most towns, fast food restaurants had similar or higher composite scores compared to sit-down restaurants. Town A had the highest composite score for sit-down restaurants (23) compared to other towns (Town B: 3.6; Town C: 10.2; Town D: 7.0). Town B, on the other hand, had both the highest composite score for fast food restaurants (18.5) as well as highest food pricing. Forty two out of 69 (60.9%) restaurants offered healthy options like main dish salads, and 33 (47.8%) offered more than 2 choices. Among those 26 offered low fat salad dressing and 21 had more than 2 choices. Few restaurants in all four towns offered a children’s menu, and only 18 offered a low-fat milk option.

**Table 3:** NEMS composite scores by restaurant types in all four towns

		Town A^a^		Town B			Town C			Town D		
	Maximum Possible Score	SD	FC	SD	FC	FF	SD	FC	FF	SD	FC	FF
Sources of Information	12.0	4.0	0.0	0.0	0.0	6.5	0.8	0.7	3.2	0.0	0.0	2.3
Site Visit Information	9.0	3.0	0.0	0.0	−1.0	1.0	−1.5	−0.9	−0.8	−0.5	0.0	−0.4
Menu Review/Site Visit	12.0	6.0	0.0	0.9	5.0	2.5	2.8	3.0	1.0	2.5	3.0	1.9
Menu Review	18.0	10.0	0.0	5.7	3.3	7.5	6.2	4.7	5.8	5.0	3.5	4.3
Facilitators and Support	9.0	6.0	0.0	0.0	0.0	2.0	1.9	0.4	1.5	0.5	0.0	0.8
Barriers	0.0	0.0	0.0	0.0	−1.0	−0.5	−6.4	−0.9	−0.8	0.0	− 1.5	− 0.4
Pricing	3.0	0.0	0.0	−3.0	−2.0	−2.0	−1.5	0.0	− 1.5	− 1.5	−1.5	− 1.1
Children’s Menu	9.0	0.0	0.0	0.0	4.5	3.8	3.3	6.0	3.8	1.2	0.0	2.0
Composite	72.0	23.0	0.0	3.6	7.3	18.5	10.2	8.0	9.8	7.0	3.5	7.6

*SD* sit down, *FC* fast causal, *FF* fast food

^a^No data is available on fast foods as there are no fast foods in this town

*NEMS* Nutrition Environment Measures Survey

The tool for NEMS for Restaurants are from Saelens et al. [11]

Data collected July–November 2013

### Perception and experience with the food environment

Thirty-two participants completed an interview. Participant characteristics are described in Table 4.

**Table 4:** Demographics characteristics of the participants from the semi-structured interviews(n = 32)

Variables	n (%)
Age (in years), mean (SD)	35.6 (6.2)^a^
Number of Children, mean (SD)	3.7 (1.6)^a^
Gender	
Male	1 (3)
Female	31 (97)
Household income	
< $15,000	6 (19)
$15,000 - $34,999	20 (62)
$35,000 - $50,000	6 (19)
Employment	
Full/Part time	18 (56)
Unemployed	14 (44)
Marital Status	
Married or living with a partner	28 (88)
Not married	4 (12)
Insurance	
Uninsured	19 (60)
Insured	13 (40)
Education	
Less than high school	18 (62)
High school diploma or GED	6 (21)
Some college or Bachelors’ degree	5 (17)
Country of Origin	
Mexic	29 (90)
U.S.-born	3 (9)
Years in the U.S.	
≤ 10	1 (3)
11 or more	28 (97)
Interview Language	
English	5 (16)
Spanish	27 (84)
BMI, mean (SD)	32.2 (5.8)^a^
Obesity Status	
Healthy weight	2 (7)
Overweight	10 (31)
Obese	20 (63)

^a^Mean and Standard Deviation

Education missing for 3 participants

Years in the US missing for 3 participants

#### Perceived accessibility of fresh produce

The majority of participants reported that fruits and vegetables were easily accessible and they did not experience difficulties purchasing them in their community. When asked what made it easy to access fruits and vegetables, many participants distinguished accessibility (ease of access to fruits and vegetables from their community) from availability (perceptions of the supply and appropriate amounts of fruits and vegetables to match community’s needs). For example, most participants mentioned that fruits and vegetables were readily available in their neighborhood; however, accessibility was heavily dependent on farming seasons as seasonality of the produce impacted pricing. One female participant stated:

“…There’s an abundance [of fruits and vegetables] and they’re not expensive or one may have family members who have fruit trees, and get them that way […] and we exchange fruits. If we work on the apple [orchards] and they in peaches or apricot, then they give us and we give them and like so.” (Lorena, 32)

Another participant emphasized the impact of seasonal produce on price:

“It’s easy and difficult at the same time because of the prices. We’re normally living paycheck to paycheck and when it’s strawberry season, well, we take advantage of it and buy strawberries because they’re on sale, cheap. Same with other fruits. All of that is easier to get at a lower price when it’s in season.” (Marissa, 33)

#### Perceived quality of produce

Most participants described the quality of produce in their neighborhood as good. When asked whether quality produce was available in their local food environment, participants said the good quality produce in the community came from both farming fields and chain grocery stores. One participant asserted, “If you want [fruits and vegetables] very fresh, you can go to those places where they sell them freshly picked. Or if not, the Safeway and Wal-Mart stores always have them anyways. They’re fresh” (Maribel, 39). Another participant explained, “of the fruits, I get the best because I get the fruit at the fields. And I cut it myself. And in regards to the vegetables, I go to the stores and that’s where I find the vegetables” (Vanessa, 37).

#### Food access and food consumption

Seasonality tended to impact food access and therefore family meals. While some participants reported that seasonality did not affect their food consumption as their menus stayed the same; others indicated that their menus were different based on their food access. Summer menus consisted of fresh salads and fruits, while winter menus were soup-based (e.g., “pozoles”) or food heavy on protein (e.g., “carne asada” “tamales”). One participant stated how access to produce drives what meals she cooks in the summer.

“Well…I try to make more meals with vegetables like fajitas, in order to try and use as many vegetables as possible and fruits, fruit water as well. Everything is freshly harvested and the vegetables and the fruits are also freshly picked.” (Lorena, 32)

Other participants shared similar sentiments of eating fresh produce and noted their ability to “plant” and “harvest” their own vegetables for consumption. Winter seasons seemed to affect food availability and food access as less produce is harvested and many are employed in agricultural industries. Participants reported being resourceful and freezing fresh vegetables from the summer to be consumed in the winter.

#### Food store preference

Most participants mentioned that their decision to shop at different stores was based on food quality, price, and nearest location to their home. Those who preferred quality food said they shopped at large grocery stores in their community. Many participants also emphasized that price was key when shopping for food, regardless of the type of stores. For example, this participant stressed the importance of price, saying, “I go to different stores, and I buy wherever the prices are cheaper. I go and I buy and if I see that it’s cheaper somewhere else, then I’ll go to the other store” (Vanessa, 37).

Geographic location was another key factor of food store preference, particularly among those who reported shopping frequently (more than once a week). When asked how they decide where to shop for food, one participant stated, “Well, whatever is closer to me” (Lorena, 32).

#### Familial behavior around eating out

Many participants reported eating out regularly between 2 and 4 times per week. They also reported eating out more regularly during the summer than winter when the lack of farm work puts constraints on their budget. They most frequented local Chinese and American buffets or fast-food chains specializing in burgers and pizza. When asked how they make decisions about where to eat out, most reported that they decided together as a family. Frequency of dining out varied across participants, wherein some preferred to eat out once per week and others less frequently due to financial constraints. Participants also explained that when eating out, their children ordered for themselves, although some added that the children must first have their parents’ consent. Illustrating this theme, one participant said of her children ordering their own food, “They do. They do, but we supervise them because if we let them, they’ll ask and they won’t eat everything (Maria, 36).” Another participant explained, “Well like I told you, it’s one day where we let them have the pleasure of eating what they want. They decide” (Alicia, 44).

## Discussion

This study found that the overall food environment quality composite scores for both food stores (grocery stores and convenience stores) and restaurants were very low, indicating the limited availability of healthier options in the food environment. There were more convenience stores than grocery stores and more sit-down restaurants compared to fast causal and fast food restaurants in four predominantly Hispanic rural farming communities.

Grocery stores had greater availability of healthier options and better quality produce compared to convenience stores. Qualitative interview results supported this finding, as participants reported having availability and access to fresh food products in chain grocery stores or farming fields. These findings corroborated results from other studies in rural communities in the US and other international setting that grocery stores offer more healthful food selections but are outnumbered by convenience stores [23–25]. Although convenience stores offer fewer varieties of healthful food choices, [16, 24–27] they are also located in more accessible areas, potentially leading to higher customer traffic of neighborhood residents [24, 27, 28]. A recent study showed that proximity helps individuals build close social ties with food store owners impacting their preference to shop in smaller food stores [25].

The price of food scores identified in NEMS among grocery stores and convenience stores were similarly low, and considerably lower than the maximum price score of 22. The low price scores indicate that the healthier options were more expensive than regular items. In one of the towns, the grocery store scored lower than the convenience store indicating that although grocery stores carry healthier options, prices are also higher. Interviews showed that food prices and geographic proximity were a big driver of store preference. Thus, if residents feel that prices are similar between grocery stores and convenience stores, residents may prioritize geographic proximity and shop at convenience stores, where fewer healthy food options are available [29]. In agricultural regions like the communities in this study, participants seem to resort to other options like accessing seasonal produce through networks of friends and families associated with farming industries and adapting their main menus based on produce availability.

Restaurants generally lacked basic practices to encourage more healthful food choices, such as offering healthy entrées and main dish salads, fruits, baked chips, and whole grains. Regardless of restaurant type, the composite scores on healthier options were very low. When compared across restaurant types for healthier options, fast food restaurants scored similarly or healthier to sit-down restaurants. This finding may not be construed as fast food restaurants offering heathier options as it may be a reflection of adherence to the changing regulations on chain restaurants such as providing nutrition information, promotion of healthier foods, lower pricing, and availability of a children’s menu [30]. Many of the sit-down restaurants in the communities were family-owned; they, therefore, may be slower to adopt new regulations, consequently impacting their overall NEMS-R score. Participants also voiced a preference for buffet typed restaurants for dining out. When faced with a large amount or wide variety of foods such as at a buffet, individuals tend to overeat and the large portion sizes contribute to obesity rates [31]. Future intervention studies may want to address portion control when eating out at restaurants and how to avoid multiple trips to buffet lines.

Although participants perceived having access to healthier options in their food environment, nearly all of the participants (94%) were overweight or obese. This finding may be indicative that rather than perception of the food environment, the objective environment may have greater impact in their resident’s health as shown in prior studies [32, 33]. The discordance between the objective environment and the individual’s perception may be a reflection of the relative change in food availability during farming vs. non-farming seasons and/or participants’ “optimism” about their life. Research on Hispanic immigrants has extensively documented their resilience, and their ability to look at life from a positive outlook when faced with challenging circumstances [34]. Rather than feeling food insecure, participants may feel they have more than what they had in their home country and mobilize social network of family and friends to get help. Future research may want to examine ways to reconcile these differences and explore whether resilience plays a role when resources are limited.

The study had several limitations. The food environment of this study may not be representative of other rural food environments as our study was based in an agricultural region and may not be translatable to other rural areas. Some components of the food environment (e.g., vending machines, worksite cafeterias) were not included. The study also excluded places that were not regularly frequented by the adult population such as school concessions. Additionally, results of NEMS represent one point in time and cannot account for previous or future alterations on restaurant menus, seasonal variations in menu or store items and price, or emergence of new and changing store format.

## Conclusion

Community members’ perception of food availability and food access are different from the objective assessment of food environment. Information gathered using mixed-methods provides an inclusive perspective that can inform community-wide interventions to address the food environment in these rural communities.

## Abbreviations

CHW: Community Health Worker
FC: Fast casual
FF: Fast food
GED: General Educational Development
NEMS: Nutrition Environment Measures Survey
NEMS-R: Nutrition Environment Measures Survey-Restaurants
NEMS-S: Nutrition Environment Measures Survey-Stores
SD: Sit down
SD: Standard deviation
